# The League of Mercy

**Published:** 1910-02-12

**Authors:** 


### THE LEAGUE OF MERCY.

### COMPLETE LIST OF THE RESULTS OF EACH DISTRICT IN 1909 COMPARED WITH 1908.

We have the pleasure to publish the following complete list of the total yield of each district in 1909, which differs materially in certain cases from the estimates we published in our issue of December 25, p. 367. It will be seen that the total income raised by the whole of the districts in 1909 was £21,486, compared with a total of £21,630 for 1908. We feel confident that the Presidents, Vice-Presidents, and members of each district will study these figures with great interest, and that such a study will lead to still further and still better results than those already reached, good as they are.

Yield of each District in 1909.

Kensington (South) £1,537, as against £1,623, a decrease of £86.
St. George's £1,045, as against £974, an increase of £71.
Paddington (South) £896, as against £775, an increase of £121.
Fulham £864, as against £931, a decrease of £67.
Berks £689, as against £727, a decrease of £38.
Hants £686, as against £675, an increase of £11.
Epsom—Esher £674, as against £581, an increase of £93.
Marylebone (West) £584, as against £659, a decrease of £75.
Sevenoaks £521, as against £453, an increase of £68.
Westminster £519, as against £318, an increase of £201.
Marylebone (East) £519, as against £615, a decrease of £96.
Kingston—Surbiton £518, as against £504, an increase of £14.
Strand £429, as against £412, an increase of £17.
Lewisham £369, as against £234, an increase of £135.
Bucks £354, as against £326, an increase of £28.
Sussex (East) £350, as against £383, a decrease of £33.
Oxford £339, as against £320, an increase of £19.
Hornsey—Finchley £336, as against £317, an increase of £19.
Kensington (North) £311, as against £394, a decrease of £83.
St. Pancras (North) £304, as against £402, a decrease of £98.
Ealing £299, as against £189, an increase of £110.
Tonbridge £296, as against £281, an increase of £15.
Deptford—Brockley £295, as against £228, an increase of £67.
Mid-Essex (Chelmsford) £293, as against £267, an increase of £26.
Hampstead £271, as against £327, a decrease of £56.
Chertsey—Woking £269, as against £270, a decrease of £1.
Hammersmith £267, as against £248, an increase of £19.
Guildford £258, as against £255, an increase of £3.
N.E. Sussex £241, as against £265, a decrease of £24.
Brentford £234, as against £297, a decrease of £63.
Chelsea £230, as against £467, a decrease of £237.
Dulwich and Norwood £226, as against £212, an increase of £14.
St. Pancras (South) £210, as against £1, an increase of £209.
Luton £209, as against £198, an increase of £11.
Eastbourne £203, as against £210, a decrease of £7.
City of London £203, as against £123, an increase of £80.
Lambeth—Brixton £202, as against £163, an increase of £39.
Epping £197, as against £191, an increase of £6.
Eye £193, as against £222, a decrease of £29.
Hertford £192, as against £236, a decrease of £44.
St. Pancras (West) £191, as against £198, a decrease of £7.
Richmond £173, as against £213, a decrease of £40.
Woolwich £167, as against £188, a decrease of £21.
Horsham £166, as against £158, an increase of £8.
Battersea £162, as against £118, an increase of £44.
S.E. Essex £158, as against £152, an increase of £6.
Uxbridge £160, as against £206, a decrease of £46.
Hitchin £146, as against £104, an increase of £42.
Ashford £142, as against £196, a decrease of £54.
Maldon £140, as against £157, a decrease of £17.
Medway £139, as against £124, an increase of £15.
Hackney (North) £135, as against £141, a decrease of £6.
Croydon £135, as against £91, an increase of £44.
Southwark—Bermondsey £135, as against £112, an increase of £23.
Romford and Ilford £133, as against £129, an increase of £4.
East Grinstead and Hayward's Heath £132, as against £166, a decrease of £34.
Stowmarket £123, as against £144, a decrease of £21.
Reigate £121, as against £137, a decrease of £16.
Greenwich £114, as against £120, a decrease of £6.
Mile End £114, as against £129, a decrease of £15.
Gravesend £107, as against £92, an increase of £15.
Enfield £100, as against £96, an increase of £4.
Finsbury (Central) £96, as against £77, an increase of £19.
Brighton—Hove £92, as against £198, a decrease of £106.
Watford £89, as against £64, an increase of £25.
Isle of Thanet £88, as against £96, a decrease of £8.
Lewes £82, as against £76, an increase of £6.
Islington (West) £75, as against £83, a decrease of £8.
Wimbledon £73, as against £72, an increase of £1.
Hythe £72, as against £80, a decrease of £8.
Whitechapel £69, as against £83, a decrease of £14.
Streatham £67, as against £78, a decrease of £11.
Colchester £63, as against £64, a decrease of £1.
Biggleswade £63, as against £102, a decrease of £39.
Limehouse £61, as against £71, a decrease of £10.
Harwich £58, as against £50, an increase of £8.
Camberwell (North) £58, as against £48, an increase of £10.
Bethnal Green £55, as against £31, an increase of £24.
Holborn £49, as against £55, a decrease of £6.
Worthing £47, as against £54, a decrease of £7.
Lowestoft £45, as against £73, a decrease of £28.
Rochester £43, as against £44, a decrease of £1.
Lambeth—Kennington £43, as against £41, an increase of £2.
Sudbury £43, as against £47, a decrease of £4.
Finsbury (East) £41, as against £42, a decrease of £1.
St. Albans £40, as against £55, a decrease of £15.
Faversham £39, as against £39, no change.
St. Pancras (East) £32, as against £25, an increase of £7.
Camberwell (Peckham) £29, as against £29, no change.
Walthamstow £28, as against £30, a decrease of £2.
Clapham £27, as against £26, an increase of £1.
Dartford £25, as against £20, an increase of £5.
Southwark (West) £24, as against £23, an increase of £1.
Saffron Walden £21, as against £25, a decrease of £4.
Paddington (North) £15, as against £227, a decrease of £212.
Chichester £7, as against £7, no change.
Willesden £2, as against *nil*, an increase of £2.

---

At the 131st annual meeting of the Governors of the London Dispensary, Mr. Deputy Barham, C.C., said that the number of patients treated at the dispensary last year was 2,581, and 580 were visited by the doctors at their own homes.

The annual meeting of the "After Care Association" for poor persons discharged recovered from asylums for the insane will be held in the Library of the Royal College of Physicians, 12 Pall Mall, S.W., on February 16, at 4 p.m. Sir R. Douglas Powell, Bart., will take the chair.

We have to record the death of Dr. Daniel Mowat. He was educated in Edinburgh, London, and Paris, and obtained with honours the M.D. degree of Edinburgh University in 1887. He was also clinical assistant at the Royal London Ophthalmic Hospital for twenty years, and at the Soho Hospital for Women. He was also consulting medical officer to the London City Mission. He was, besides, a major of the R.A.M.C., commanding officer of the Third East Anglian Field Ambulance, and a lecturer on ambulance work. He was a Fellow of the Royal Society of Medicine and a member of the Ophthalmological Society.

